# Genome-Wide Identification of microRNAs Associated with Starch Biosynthesis and Endosperm Development in Foxtail Millet

**DOI:** 10.3390/ijms25179282

**Published:** 2024-08-27

**Authors:** Qiang Li, Dongming Li, Shihua Guo, Xiaofang Yu

**Affiliations:** 1 College of Agriculture, Inner Mongolia Agricultural University, Hohhot 010000, China; liqiang@emails.imau.edu.cn; 2 Key Laboratory of Crop Cultivation and Genetic Improvement in Inner Mongolia Autonomous Region, College of Agronomy, Inner Mongolia Agricultural University, Hohhot 010000, China; 3 Inner Mongolia Autonomous Region College Crop Germplasm Resources Protection and Utilization Engineering Research Center, Inner Mongolia Agricultural University, Hohhot 010000, China; 4 Key Laboratory of Herbage & Endemic Crop Biology, Ministry of Education, School of Life Sciences, Inner Mongolia University, Hohhot 010000, China; lidongming@lzu.edu.cn

**Keywords:** foxtail millet, starch, microRNA, mRNA

## Abstract

Foxtail millet is one of the oldest crops, and its endosperm contains up to 70% of starch. Grain filling is an important starch accumulation process associated with foxtail millet yield and quality. However, the molecular mechanisms of grain filling in foxtail millet are relatively unclear. Here, we investigate the genes and regulated miRNAs associated with starch synthesis and metabolism in foxtail millet using high-throughput small RNA, mRNA and degradome sequencing. The regulation of starch synthesis and quality is carried out mainly at the 15 DAA to 35 DAA stage during grain filling. The DEGs between waxy and non-waxy foxtail millet were significant, especially for *GBSS*. Additionally, ptc-miR169i_R+2_1ss21GA, fve-miR396e_L-1R+1, mtr-miR162 and PC-5p-221_23413 regulate the expression of genes associated with the starch synthesis pathway in foxtail millet. This study provides new insights into the molecular mechanisms of starch synthesis and quality formation in foxtail millet.

## 1. Introduction

Foxtail millet [*Setaria italica* (L.) P. Beauv.], a diploid (2n = 2x = 18) annual species, is one of the oldest cereals [1]. It is an important global millet crop and has been cultivated since the period of ancient civilizations as a staple [2]. Moreover, it is an essential food and feed crop in some arid and semi-arid regions, and is widely grown in Asia and Africa, especially in China and India. Foxtail millet has become an excellent model plant for studying C4 monocots and has been widely utilized for research on plant growth and development [3]. Starch is an important human energy source and is also an important foxtail millet component, comprising about 70% of foxtail millet, and starch biosynthesis is of crucial importance since starch content determines the yield, quality, and economic value of foxtail millet [4]. Starch fine structure and physicochemical properties are influenced by multiple elements such as genetics and environmental conditions. Starch in foxtail millet comprises amylose and amylopectin, and their ratio directly affects cooking quality. Foxtail millet is divided into three types, relative to amylose content (AC): non-waxy (AC: 0~3.5%), low-AC (AC: 7.8~16%), and waxy (AC: 17~31.9%); the amylose content in many cereals is mainly controlled by the *GBSS I* gene [5]. Several genes associated with starch synthesis have been identified, including granule-bound starch synthase (*GBSS*), ADP-glucose pyrophosphorylase (*AGPase*), soluble starch synthase (*SSs*), starch branching enzyme (*SBE*), starch debranching enzyme (*DBE*), glucan-branching enzyme (*GBE*), and UDP-glucose pyrophosphorylase (*UGPase*) [6,7,8]. Although the functions of these genes have been well characterized, reports on these genes on starch synthesis and accumulation during grain filling are rare in foxtail millet.

MicroRNAs (miRNAs) are small (18–25 nt), and they are a class of endogenous non-coding RNAs with regulatory functions [9]. Mature miRNAs recognize target mRNAs through base complementary pairing, degrade target mRNAs, or inhibit their translation [10]. The miRNAs are involved in grain development [11]. The miRNAs in several crop species play a crucial starch synthesis role during grain development and filling. For example, in maize (*Zea mays*), at the post-transcriptional level, high expression of Zma-miR159k-3p negatively regulates *ZmMYB138* and *ZmMYB115*, which in turn affects the transcriptional activity of *Du1/Wx* and *Ae1/Bt2* genes in their respective promoter regions [12]. The miR172 regulates the expression of genes that inhibit starch biosynthesis in citrus [13]. The down-regulation of *NnSS2* expression by NnumiR396a resulted in the prevention the synthesis of amylopectin [14]. The miR160-, miR164- and miR390- mediated auxin signaling pathways were identified as regulating grain filling [15]. Although several studies have investigated starch synthesis mechanisms in staple crops, few have explored miRNAs involvement in starch synthesis in foxtail millet.

To date, there have been few reports using integrated high-throughput sequence data to reveal more about the mRNA and miRNA regulation of starch synthesis in foxtail millet. Therefore, we used transcriptome, miRNA and degradome sequencing to identify mRNAs and miRNAs and their target genes involved in starch synthesis regulation in waxy and non-waxy foxtail millet. Our study provides insights into the roles of mRNAs and miRNAs associated with starch synthesis and metabolism and will accelerate the breeding of new foxtail millet cultivars with improved quality.

## 2. Results

### 2.1. Phenotyping and Amylose Determination

**Figure 1:**
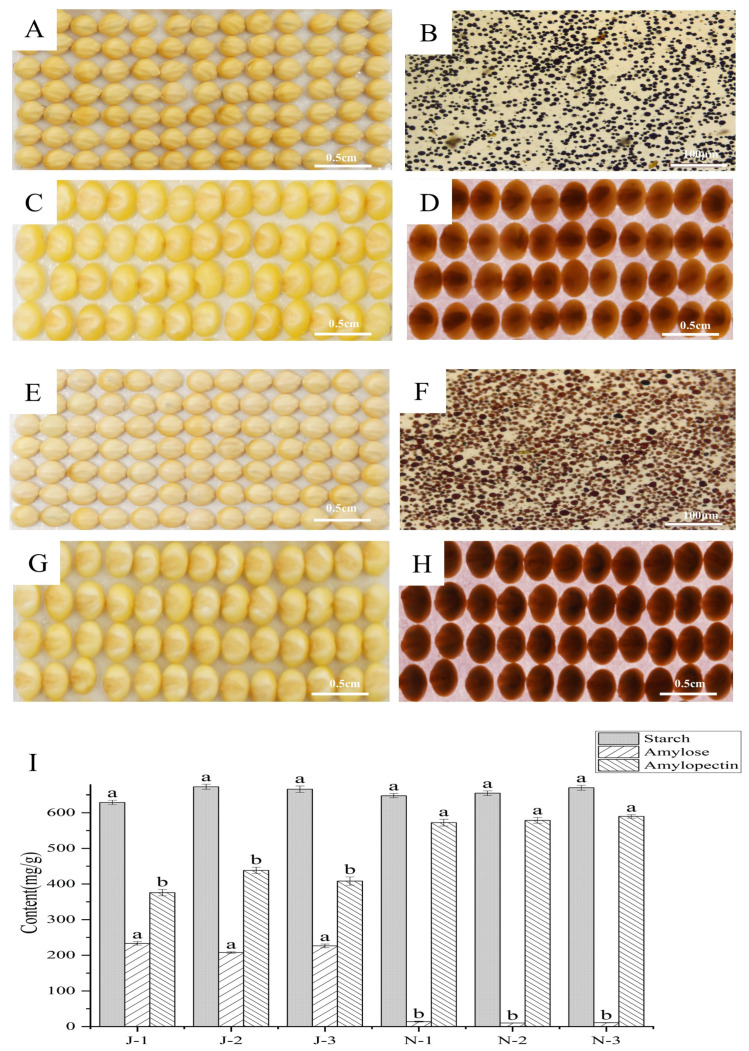
Different phenotypes, starch content, and characteristics between waxy and non-waxy seeds. (**A**) Characterization of seed appearance in J (non-waxy). (**B**) Characterization of seeds via I_2_/KI staining of J. (**C**) Characterization of J seeds after shell removal. (**D**) Transmission of J shelled seeds. (**E**) Characterization of seed appearance in N (waxy). (**F**) Characterization of seeds via I_2_/KI staining of N. (**G**) Characterization of N seeds after shell removal. (**H**) Transmission of N shelled seeds. (**I**) Content of amylose, amylopectin, and starch of J and N, different letters represent differences at the *p* < 0.05 significance level.

The CG4 (♀) × MZN (♂) and MZN (♀) × CG4 (♂) F_1_ and F_2_ generation phenotypes were characterized using I_2_/KI staining. The F_1_ products of both combinations were non-waxy and the separation ratio of waxy: non-waxy was 1:3 in the F_2_ population (Supplementary Table S2). Waxy and non-waxy foxtail millet were selected from the recombinant inbred line (RIL) population of MZN (♀) × CG4 (♂). The appearance of the two materials was comprehensively evaluated from multiple aspects, and by comparing the seeds of the two materials, there was a significant difference in the glossy color (Figure 1A,E). Removal of the glumes revealed significant differences in the color and transparency of waxy and non-waxy material seeds, with non-waxy seeds being more transparent than waxy ones (Figure 1C,D,G,H). The phenotypes of both materials were analyzed. The non-waxy starch turned dark blue, while the waxy starch turned reddish-brown (Figure 1B,F). To determine material starch content and structure differences, starch, amylose, and amylopectin content (Figure 1I) were determined from mature seeds. Starch content was about 700 mg/g in waxy and non-waxy foxtail millet, amylose content was significantly higher for non-waxy than waxy millet, while non-waxy foxtail millet lacked amylopectin.

### 2.2. Morphological Characteristics of Starch

**Figure 2:**
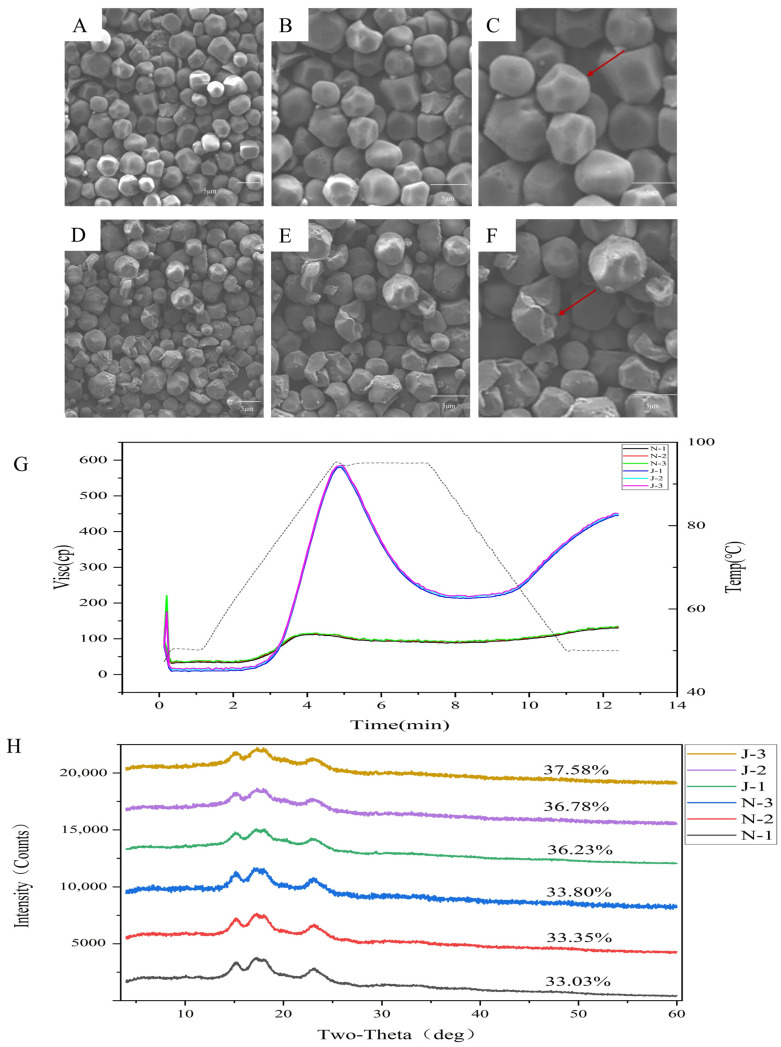
(**A**–**C**) Scanning electron microscope microstructure of starch in non-waxy foxtail millet, the red arrow represents the typical of starch granules. (**D**–**F**) Scanning electron microscope microstructure of starch in waxy foxtail millet. (**G**) Pasting properties of non-waxy and waxy foxtail millet. (**H**) XRD patterns and relative crystallinity (RC) of non-waxy and waxy foxtail millet.

Foxtail millet starch scanning electron microscope images showed that non-waxy starch granules were irregularly polygonal, with full starch granules and smooth and structurally intact surfaces (Figure 2A–C). Conversely, waxy starch granules were wrinkled, and many granules showed numerous breakages (Figure 2D–F). This may be have been due to the different proportions of amylose and amylopectin: non-waxy grains have high amylose content and are more complete, whereas waxy grains have almost no amylose content and a high proportion of amylopectin, resulting in lower mechanical strength and susceptibility to breakage.

### 2.3. XRD Patterns and Pasting Properties of Starch

The crystalline structures of waxy and non-waxy grain starches were analyzed using an X-ray diffractometer (Figure 2H). The X-ray diffraction spectra of all starch samples were similar, with four major diffraction peaks near 15, 17, 18, and 23° (2θ). The non-waxy grains had a higher degree of crystallinity than the waxy grains, and starch granules with low straight-chain amylose content had a higher degree of crystallinity than normal starch granules. The decrease in amylose content may have been a major reason for the increase in the degree of crystallinity.

The non-waxy types PV, TV, and FV were approximately 114, 89, and 132 centipoises, respectively. The peak, trough, and final viscosities of waxy foxtail millet were only 19.56, 41.52 and 29.58% of those of non-waxy foxtail millet. This is because of the higher amylose content of non-waxy foxtail millet (Figure 2G and Supplementary Table S3).

### 2.4. Summary of Transcriptome Data during Grain Filling in Foxtail Millet

Transcriptome analysis of developing endosperm revealed that, after removing low-quality reads from the raw data, 104.53 Gb of valid data was obtained (Supplementary Table S4). We conducted transcriptome analysis of differentially expressed genes (DEGs) at the foxtail millet developing endosperm grain filling stage. A total of 6911 DEGs were obtained in the three comparison groups, of which 3796 genes were up-regulated and 3115 genes were down-regulated (Supplementary Figure S4). The ‘J_1dVSN_1d’ comparison group included 125 up-regulated genes and 140 down-regulated genes, the ‘J_15dVSN_15d’ comparison group included 1994 up-regulated genes and 1187 down-regulated genes, and the ‘J_35dVSN_35d’ comparison group included 1677 up-regulated genes and 1788 down-regulated genes. 

Gene ontology (GO) term enrichment analysis suggested that the DEGs be separated into three categories: ‘biological process (BP)’, ‘cellular component (CC)’ and ‘molecular function (MF)’. Gene ontology (GO) term enrichment analysis showed that DEGs between waxy and non-waxy foxtail millet were significantly enriched in cellular components such as ‘nucleus’, ‘cytoplasm’ and ‘membrane’ at 1 DAA and 15 DAA. At 35 DAA, the DEG enrichment analysis identified cellular components such as ‘nucleus’ and ‘chloroplast’ (Supplementary Figure S2A–C). Additionally, Kyoto Encyclopedia of Genes and Genomes (KEGG) pathway enrichment analysis suggested that the DEGs were located in protein processing in ‘Plant-pathogen interaction Cutin’ between waxy and non-waxy millet at 1 DAA, 15 DAA and 35 DAA (Supplementary Figure S2D–F). Notably, at the metabolic level, ‘starch and sucrose metabolic’ contents were enriched in a large number of differential genes in three periods, especially 15 DAA and 35 DAA.

**Figure 3:**
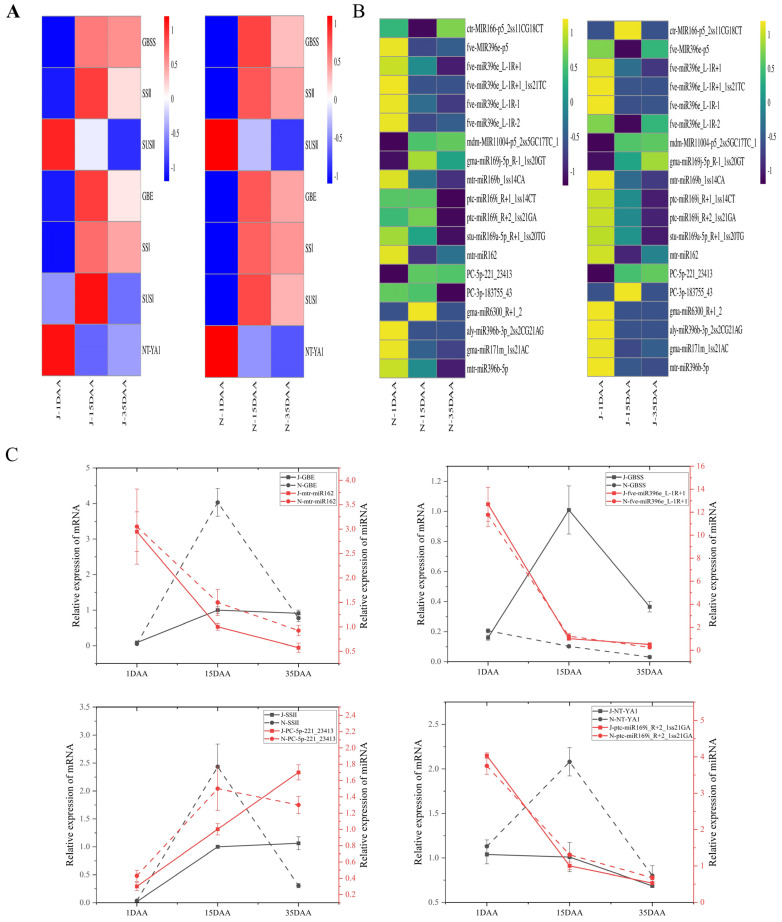
(**A**) Key genes related to starch synthesis of waxy and non–waxy foxtail millet. (**B**) miRNAs related to the starch synthesis of waxy and non-waxy foxtail millet. (**C**) Expression pattern of key miRNAs and their target genes.

Starch plays an important role in grain filling development. The numerous DEGs related to starch synthesis were enriched in the starch and sucrose metabolism pathways during endosperm development in foxtail millet (Figure S1A). Comparing the differentially expressed genes in the starch and sucrose metabolic pathways for each of the comparison groups, we found that (Figure 3A and Figure S1B,D) 1,4-alpha-glucan-branching enzyme (Seita.1G179000, *GBE*), granule bound starch synthase (Seita.4G022400, *GBSS*), soluble starch synthase 1 (Seita.4G065500, *SSI*) and sucrose synthase 1 (Seita.4G041500, *SUSI*) had similar expression trends. Expression levels of these genes increased from 1 DAA to 15 DAA, and decreased from 15 to 35 DAA. *GBSS* expression was highest at 15 DAA and lowest at 1 DAA during the entire grain development period, and its expression in non-waxy millet was significantly higher than that in waxy foxtail millet at 15 DAA and 35 DAA. 

### 2.5. Identified Differential Expression of miRNAs in Developing Endosperm

To illustrate the role of miRNAs in grain development regulation, waxy and non-waxy foxtail millet spikelets were harvested at 1 DAA, 15 DAA and 35 DAA. A total of 204,902,545 raw reads were assembled from 18 sRNA libraries via miRNA sequencing (Supplementary Table S5). Unique miRNA lengths ranging from 18 to 25 nt were obtained by analyzing and filtering the raw data. Of these, 21 nt accounted for 26.2%, and 24 nt accounted for 50%, respectively (Supplementary Figure S3). 

We screened for differentially expressed miRNAs using *p* < 0.1 as a threshold. A total of 46 differentially expressed miRNAs were identified at 1 DAA (Supplementary Table S6 and Figure S1C). Among these, 30 were up-regulated, and 16 were down-regulated. Seventeen known miRNAs were dispersed in 13 miRNA families, and 29 previously unknown miRNAs were identified. Of the 17 known miRNAs, 13 were up-regulated, and 4 were down-regulated. Of the 29 previously unknown miRNAs, 17 were up-regulated, and 12 down-were regulated. Other miRNAs were detected in waxy or non-waxy foxtail millet, including PC-3p-78123_153, PC-3p-210954_34 and PC-3p-266660_22 at 1 DAA in waxy foxtail millet. A total of 74 differentially expressed miRNAs were identified at 15 DAA (Supplementary Table S7 and Figure S1C). Among these, 31 were up-regulated, and 43 were down-regulated. Forty-four known miRNAs were dispersed in 22 miRNA families, and 30 previously unknown miRNAs were identified. Among the 44 known miRNAs, 18 miRNAs were up-regulated and 26 were down-regulated, while mtr-MIR167b-p3_2ss12GT21CT, mtr-miR166a_L+1R-1 and mtr-miR172c-5p_2ss2TC7TC were up-regulated at least one-fold. Of the 30 previously unknown miRNAs, 13 were up-regulated, 17 were down-regulated, and PC-3p-201769_37 and PC-3p-61614_212 were up-regulated at least three-fold. Some new miRNAs were found, such as PC-3p-140062_66, PC-5p-4326_2827 and PC-5p-224869_30, detected at 15 DAA in non-waxy foxtail millet. A total of 90 differentially expressed miRNAs were identified at 35 DAA (Supplementary Table S8 and Figure S1C). Among them, 56 were up-regulated, and 34 were down-regulated. In total, 64 known miRNAs were dispersed in 25 miRNA families, and 26 previously unknown miRNAs were identified. Of the 64 known miRNAs, 33 were up-regulated, and 31 were down-regulated. Among the upregulated miRNAs, ssl-MIR166a-p5_1ss12TA, mtr-MIR171b-p5_1ss21TC and gma-miR6300_R+6 were up-regulated at least three-fold and had the highest expression increase. Of the 15 previously unknown miRNAs, 14 were up-regulated, and only one was down-regulated in non-waxy millet compared with waxy foxtail millet, while PC-3p-131819_72 and PC-3p-100632_107 were up-regulated at least three-fold. We conducted integrated analysis based on transcriptome, miRNA and degradome sequencing, which showed that (Figure 3B and Supplementary Table S9) fve-miR396e_L-1R+1 can target *GBSS*, mtr-miR162 can target *GBE*, PC-5p-221_23413 can target *SSII*, and ptc-miR169i_R+2_1ss21GA can target *NF-YA1* (Seita.9G129400, nuclear transcription factor Y subunit A-1).

## 3. Discussion

Foxtail millet is an important grain crop for food security and sustainable agricultural development in Asia and Africa [3,16]. Starch is the main element of foxtail millet, and seed filling is an important process of grain starch accumulation, which is related to grain yield and quality [17,18]. In recent decades, starch synthesis mechanisms have been studied in staple crops, such as rice (*Oryza sativa*) [19] and wheat (*Triticum aestivum*) [20]. The starch synthesis process is relatively unclear in foxtail millet. In this study, we found that starch phenotypes, fine structure and physicochemical properties were significantly different between waxy and non-waxy foxtail millet. There were increasing trends in the content of amylose, starch, and amylopectin with grain development in waxy and non-waxy foxtail millet (Supplementary Figure S6). The different proportions of amylose and amylopectin play an important role, and they are crucial for determining quality and yield in foxtail millet. Furthermore, improving grain yield and quality is based on enhanced understanding of the regulatory mechanisms associated with grain starch synthesis. Previous reports suggested that miRNAs regulate crop starch synthesis [12]. However, the molecular mechanisms of starch synthesis are relatively unclear in foxtail millet. Therefore, we used high-throughput sequencing to identify key genes and miRNAs and their expression during grain starch synthesis, and further analyzed regulatory mechanisms during starch synthesis in waxy and non-waxy foxtail millet.

**Figure 4:**
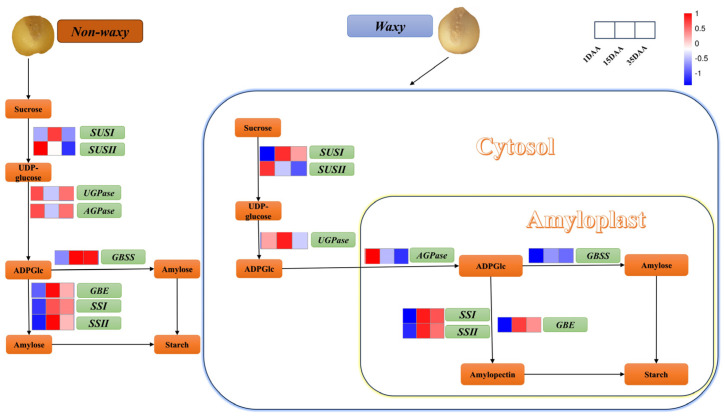
Expression profiles of starch biosynthesis genes and miRNA. Green rectangles are genes, red rectangles are up—regulated genes and blue rectangles are down—regulated genes.

Endosperm formation is initiated via double fertilization in angiosperms [21]. Endosperm development in cereals is divided into early development, differentiation and maturation [22]. In most cereal crops, about 70% of the total weight and most of the intracellular space is occupied by starch [23,24]. Starch fine structure and physicochemical properties influence endosperm quality. Amylose and amylopectin are the two main macromolecular starch components found in cereals. Amylose is a linear molecule composed of glucosyl monomers, while amylopectin has a more complex structure [25]. Starch synthesis is closely associated with gene expression. In this study, the transcriptome analysis revealed numerous genes related to starch synthesis from 15 DAA to 35 DAA during endosperm development in foxtail millet. Therefore, this stage is crucial for starch synthesis in foxtail millet. Additionally, the DEG expression between waxy and non-waxy foxtail millet was significant, especially for *GBSS* (Figure 4), which is consistent with trends reported in rice [26] and wheat [27]. 

**Figure 5:**
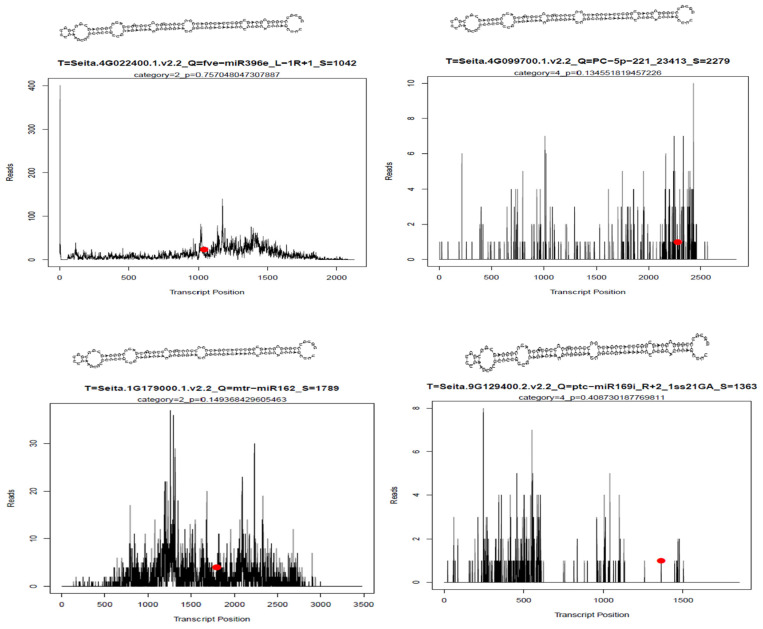
Binding site of miRNA to the target gene, the red dot indicates the cleavage site.

**Figure 6:**
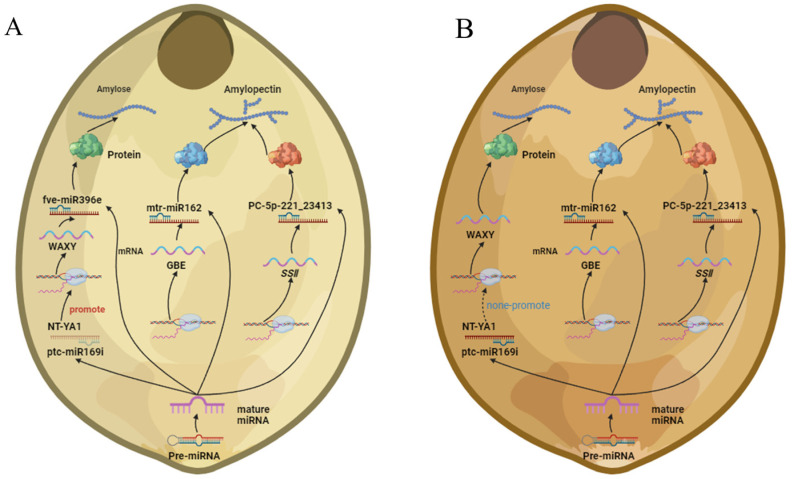
(**A**) miRNAs related to starch synthesis of non-waxy foxtail millet. (**B**) miRNAs related to starch synthesis of waxy foxtail millet. Figures created with BioRender accessed on 8 March 2024. (www.BioRender.com).

MicroRNAs (miRNAs) are a family of small non-coding RNAs that can post-transcriptionally modify gene expression through sequence complementarity [28]. Plant miRNA is involved in plant growth and development processes through the interaction between miRNA and its target gene [29,30]. Previous research has indicated that miRNAs in various crop species play a critical role in starch synthesis during grain development and filling. For example, Zma-miR159k-3p [12], miR172 [13], miRn45-5p [14], miR160-, miR164- and miR390- [15] regulate the expression of genes that affect starch biosynthesis. However, few miRNAs have been involved in starch synthesis in foxtail millet. In this study, we found in foxtail millet that miRNAs can regulate the expression of genes associated with the starch synthesis pathway. Notably, miRNA regulation of starch synthesis-related gene expression is different in waxy and non-waxy foxtail millet. For example, fve-miR396e_L-1R+1 can target *GBSS* in non-waxy foxtail millet (Figure 3C, Figure 5 and Figure 6), but we observed low levels of *GBSS* expression in waxy foxtail millet and found no evidence of miRNA regulation of *GBSS* expression. Thus, we speculated that miRNAs restrict the expression of the *GBSS* gene to some extent, preventing its uncontrolled expression, and that the *GBSS* gene is the main determinant of amylose synthesis in foxtail millet. This may represent a new regulatory mechanism for starch synthesis in foxtail millet. *NF-YA1* is able to combine the G-BOX of the *GBSS* promoter to activate gene expression and transcription [31], and we found that ptc-miR169i_R+2_1ss21GA could target *NF-YA1*. *NF-YA1* expression activated high *GBSS* expression at 15 DAA and 35 DAA in non-waxy foxtail millet, and yet ptc-miR169i_R+2_1ss21GA expression degraded *NF-YA1*, leading to a decrease in *GBSS* gene expression in waxy foxtail millet, suggesting that it may be one of the important causes of waxy and non-waxy endosperm development. We found that mtr-miR162 could target *GBE* and that its expression in 15 DAA was higher than 35 DAA in waxy and non-waxy foxtail millet, which was negatively regulated by *GBE*, suggesting that *GBE* expression can be regulated to regulate amylopectin synthesis. The newly identified miRNA PC-5p-221_23413 could target *SSII*. Starch is an important foxtail millet component. Starch fine structure and physicochemical properties influence the quality and economic value of foxtail millet, especially amylose content. Previous research initiatives have focused mainly on the *GBSS* gene effect of amylose synthesis in foxtail millet [5]. In our research, we found that miRNAs play a key role in regulating foxtail millet starch synthesis. Hence, there might be preserved miRNA-mRNA modules involved in the starch biosynthesis of foxtail millet. Moreover, we analyzed the relationship between miRNAs and known starch biosynthesis genes and constructed a complex network of miRNA-targeted starch biosynthesis regulation (Supplementary Figure S5). Nineteen microRNAs can target six genes to regulate endosperm starch biosynthesis, which reveals the mechanism of miRNA-mediated regulation of starch biosynthesis during grain endosperm development. However, the regulatory relationships and interactions between miRNAs-mRNAs in this regulatory network need to be further investigated.

## 4. Materials and Methods

### 4.1. Plant Materials

The foxtail millet cultivars ‘Chigu 4’ (CG4) and ‘Maozhuanian’ (MZN) were obtained from the College of Agriculture, Inner Mongolia Agricultural University (Hohhot, Inner Mongolia Autonomous Region, China). CG4 (non-waxy) and MZN (waxy) were used to construct F_1_, F_2_ (404 foxtail millet plants), and RIL groups (404 foxtail millet plants). The obtained F_1_ and F_2_ populations were used for the genetic analysis. We chose non-waxy (J) and waxy (N) materials from the RIL population of MNZ (♀) × CG4 (♂). They were planted in a field at the Inner Mongolia Agricultural University experimental station in 3 × 3 m^2^ plots in a randomized group design with three replications. Endosperm tissues were harvested from N and J materials at 1, 15 and 35 days after anthesis (DAA). Samples were taken from three biological replicates.

### 4.2. Phenotypic Evaluation

Waxy and non-waxy mature seeds were stained and identified using I_2_/KI. The seeds were washed and soaked in water for 24 h, and then stained with iodine/potassium iodide (I_2_/KI; 1% iodine and 3% potassium iodide) using a dissection microscope (Olympus, Tokyo, Japan). Starch, amylose and amylopectin content were measured with a kit (Sangong Biotechnology, Shanghai, China).

### 4.3. Determination of Starch Crystal Structure, Morphological Characteristics and Pasting Properties

Starch was extracted via the alkaline method [32]. Briefly, an appropriate sample of hulled foxtail millet seeds was pressed, soaked in an appropriate amount of 0.2% sodium hydroxide solution for 12 h, filtered, and shaken for 2 h. Centrifugation and scraping were repeated until the black tail layer at the top of the starch was negligible, then washed with ultrapure water 2~3 times. After centrifugation, the supernatant was poured off, dried, collected and filtered through a 100-mesh sieve. The morphological characteristics of starch analysis were determined using a scanning electron microscope (SU8100, Hitachi, Tokyo, Japan). X-ray diffraction analysis (Dmax-2200PC, Rigaku Corporation, Tokyo, Japan) was employed to determine the crystalline characteristics of the starch sample as previously described [33]. The starch pasting properties were measured using Rapid Visco Analyser (RVA 4500, Perten, Hagersten, Sweden). The trough viscosity (TV), peak viscosity (PV), final viscosity (FV), setback viscosity (SV), breakdown viscosity (BV), and pasting temperature (PT) were subjected to an evaluation process using the methodology described by Wang et al. [34]. 

### 4.4. Transcriptome and Small RNA Library Construction and Sequencing 

The eighteen non-waxy (J) and waxy (N) libraries were extracted. Total RNA extraction was conducted using TRIzol Reagent (Thermo Fisher Scientific, Waltham, MA, USA) according to the manufacturer’s instructions. The total RNA sample exhibiting an RNA integrity number of at least 7.0 was utilized in the construction of the sequencing library. The sRNA-seq, RNA-seq and degradome libraries were constructed and sequenced by LC Biology (Hangzhou, China). We removed data including those containing adapter and low-quality reads to obtain clean data via in-house Perl scripts. Sequence quality was then checked using FastQC v0.11.9. Clean data were aligned with the reference genome (https://phytozome-next.jgi.doe.gov/info/Sitalica_v2_2, accessed on 3 November 2023) using Hisat2 v2.0.5 and fragments per kilobase million reads (FPKM) for estimating gene expression levels [35]. DEG analysis of groups was performed using the DESeq2 v1.0.19 [36], where FDR < 0.05 and |Fold Change| ≥ 2 was performed with a threshold employed to identify DEGs [37]. Differential gene expression analysis was conducted using the GO and KEGG pathway methods. We conducted miRNA data analysis using ACGT101-miR (v4.2) to remove low-quality reads to obtain clean data. The miRNAs were analyzed via Blastn using miRbase22.1. RNAfold v2.4.14 software was used to compare unmapped sequences with sequences predicting hairpin RNA structures based on contiguous 120 nt sequences. A threshold of *p* < 0.1 was applied to determine differentially expressed miRNAs. Degradome sequencing was analyzed using CleaveL 3.0 [38], and mRNA corresponding to the degradome sequence was found using the Oligomap short-reading-frame calibrator. All sequences matching those in the provided small RNA library were exported using the EMBOSS software v6.5.7.0 package, and the construction of t-plots was performed using transcriptome data for the highly efficient analysis of potential miRNA targets. The targets of miRNAs exhibiting differential expression were identified as potential target genes [39]. Based on co-expression analysis, Cystoscope software (v.3.10.1) was used to construct a molecular regulatory network model of miRNA target starch biosynthesis during foxtail millet endosperm development.

### 4.5. Quantitative Real-Time PCR

Candidate miRNAs and their targets were subjected to RT-qPCR assays to assess the reliability of the expression patterns as previously described [40]. Total RNA was isolated from foxtail millet seeds using TRIzol reagent (Tiangen, Beijing, China). All primers for target mRNAs and specific stem–loop RT primers for miRNAs were designed in Primer 5.0 software (Supplementary Table S1). The reference gene was selected from Actin and U6. The relative expression of genes in different samples was determined using the 2^−ΔΔCt^ method.

## 5. Conclusions

Overall, we analyzed the starch synthesis mechanism in waxy and non-waxy foxtail millet by phenotype, mRNA and miRNA. In foxtail millet, the 15 DAA to 35 DAA stages are important for starch synthesis. The DEGs between waxy and non-waxy foxtail millet were significant, especially for GBSS. In foxtail millet, ptc-miR169i_R+2_1ss21GA, fve-miR396e_L-1R+1, mtr-miR162 and PC-5p-221_23413 can regulate the expression of genes associated with the starch synthesis pathway. Additionally, we revealed candidate genes and miRNAs involved in starch synthesis, which may provide insights into its regulation in foxtail millet. Thus, this study provides new perspectives on the starch synthesis mechanism in foxtail millet.

## Data Availability

Data are contained within the article and Supplementary Materials.

## Supplementary Materials

The supporting information can be downloaded at https://www.mdpi.com/article/10.3390/ijms25179282/s1.
