# Supplementary material for: Genome-Wide Identification of microRNAs Associated with Starch Biosynthesis and Endosperm Development in Foxtail Millet
Source: Int J Mol Sci. 2024 Aug 27;25(17):9282. doi: 10.3390/ijms25179282 (PMC11395324; doi:10.3390/ijms25179282)
Supplement: Supplementary file 1 [file ijms-25-09282-s001.zip › Supplementary Figures S1-S6.pdf]

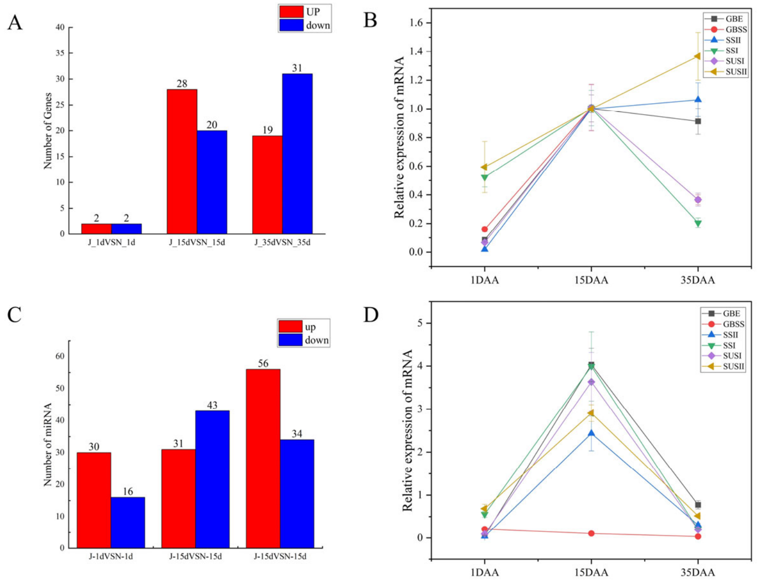

Figure S1. (**A**) The number of differentially expressed genes related to the starch and sucrose metabolism pathways. (**B**) Expression pattern of key genes in waxy foxtail millet. (**C**) The number of differentially expressed miRNAs. (**D**) Expression pattern of key genes in non-waxy foxtail millet.

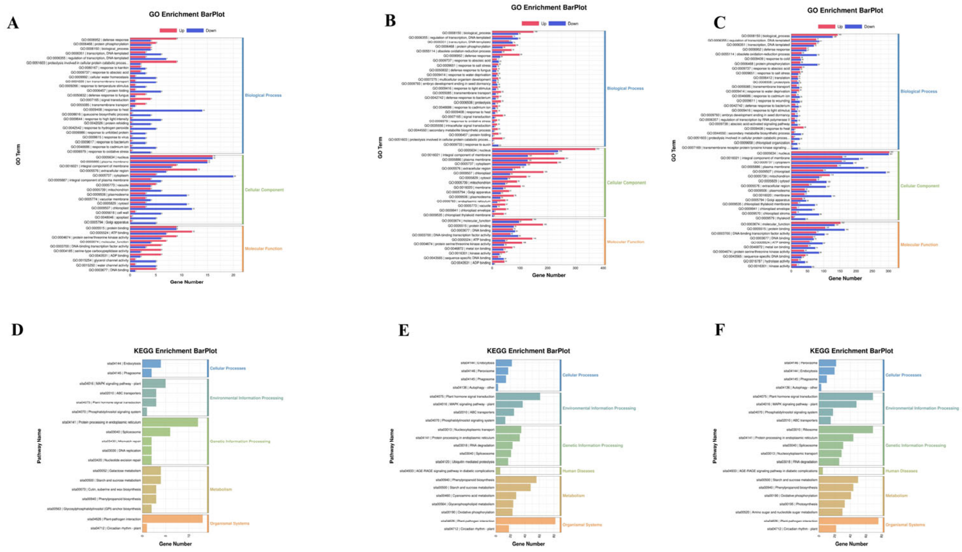

Figure S2. Go enrichment analysis of target genes of differentially expressed genes on pairwise comparisons. (**A**) 'J_1dVSN_1d'. (**B**) 'J_15dVSJ_15d'. (**C**) 'J_35dVSN_35d'. KEGG pathway enrichment analysis of target genes of differentially expressed genes on pairwise comparisons. (**D**) 'J_1dVSN_1d'. (**E**) 'J_15dVSJ_15d'. (**F**) 'J_35dVSN_35d'.

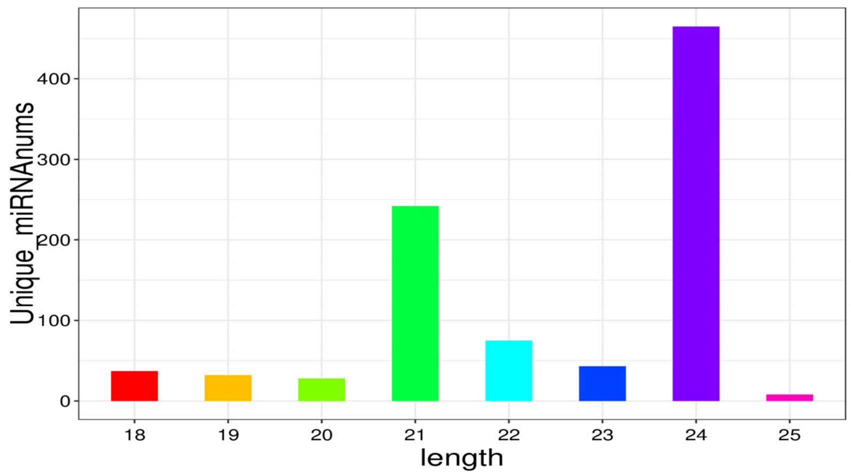

Figure S3. Length of unique miRNAs.

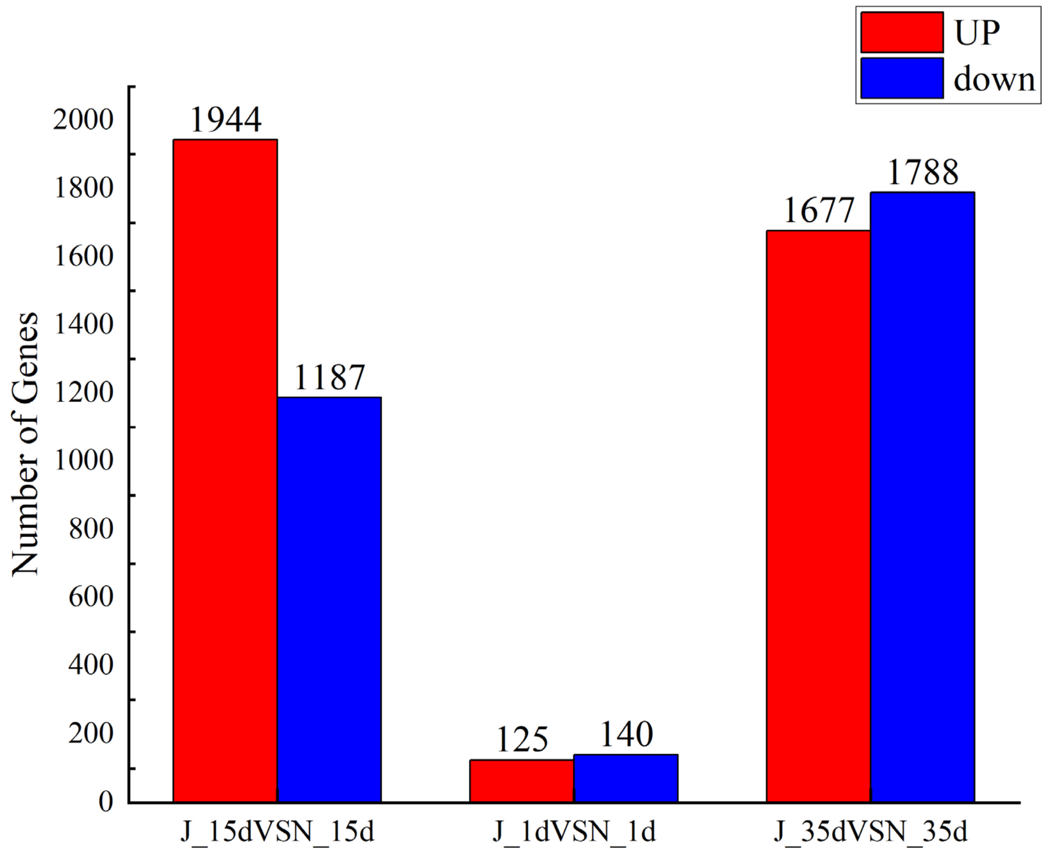

Figure S4. The number of differentially expressed genes. Red represents up-regulated significantly differentially expressed genes, and blue represents down-regulated significantly differentially expressed genes.

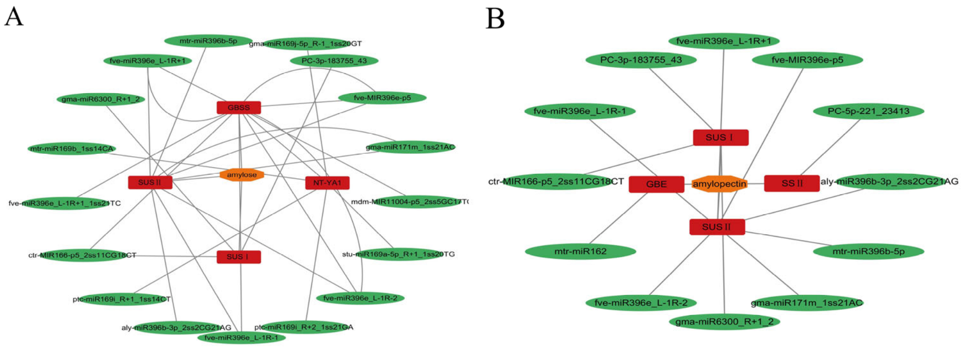

Figure S5. Co-expression network of miRNA-target-starch biosynthesis, green boxes represent miRNAs, red boxes represent target genes, and orange boxes represent amylose and amylopectin.

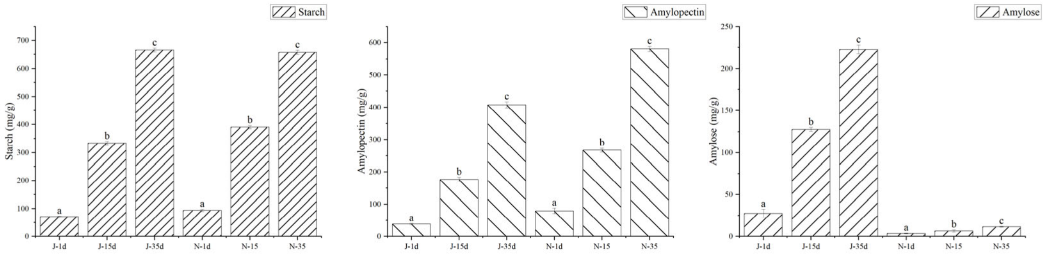

Figure S6. The content of amylose, starch, and amylopectin by grain development in waxy and non-waxy foxtail millet.
