# Supplementary material for: Genome-Wide Identification of microRNAs Associated with Starch Biosynthesis and Endosperm Development in Foxtail Millet
Source: Int J Mol Sci. 2024 Aug 27;25(17):9282. doi: 10.3390/ijms25179282 (PMC11395324; doi:10.3390/ijms25179282)
Supplement: Supplementary file 1 [file ijms-25-09282-s001.zip › Supplementary Tables S1-S9.pdf]

Table S1. A list of primers used for validation in RT-qPCR.

Type	ID	Seq
miRNA	U6-F	GGAACGATACAGAGAAGATTAGCA
	fve-miR396e_L-1R+1-F	TCCACAGGCTTTCTTGAACTG
	ptc-miR169i_R+2_1ss21GA-F	TAGCCAAGGATGACTTGCCTACA
	mtr-miR162-F	TCGATAAACCTCTGCATCCAG
	PC-5p-221_23413-F	CGATATAGCTCGGTGGCGTGTCTG
gene	SiACTIN7-F	GGCAAACAGGGAGAAGATGA
	SiACTIN7-R	GAGGTTGTCGGTAAGGTCACG
	granule bound starch synthase-F	ACTAAACCAACGCCTCCTTCC
	granule bound starch synthase-R	ATCACCAACACGAGCATACAAA
	Soluble starch synthase 2-F	GCAGTAGTGGATGTTGTGTGGA
	Soluble starch synthase 2-R	ACAACAGCGAGCCGTCACT
	sucrose synthase 2-F	TGGCAAAGTTTCAATAGTGTGG
	sucrose synthase 2-R	GGTCAGCACTCAGCAGAGAAAA
	1,4-alpha-glucan-branching enzyme 2-F	ATAATCCTCTCCCCTTCTCTGC
	1,4-alpha-glucan-branching enzyme 2-R	TAAGTCCGTCGTTCTCGCC
	soluble starch synthase 1-F	TGATGAAGAATAGGGGTTGAGG
	soluble starch synthase 1-R	GTGAGTTCTTTGGCAATGGGT
	sucrose synthase 1-F	CGGCTGTGATCTATCAATCGTA
	sucrose synthase 1-R	CAACACCTACGAGAGACAGCAA
	NF-YA1-F	TGATATTGCAGCCCTACCTTCA
	NF-YA1-R	TGTAGAAGCAGGGTGTCAGGAA

Table S2. Phenotypic genetic analysis.

Cross combination	Phenotype of F_1	Phenotype 1 of F_2	Phenotype 2 of F_2	$\chi 2$
chigu4×maozhuanian	200(non-waxy)	154(non-waxy)	46(waxy)	0.43
maozhuanian×chigu4	204(non-waxy)	147(non-waxy)	57(waxy)	0.94

Table S3. Pasting properties of non-waxy and waxy foxtail millet.

sample	Peak 1	Trough 1	Breakdown	Final Visc	Setback	Peak Time	Pasting Temp
N-1	112	88	24	130	42	4.07	62.70
N-2	114	90	24	133	43	4.07	62.70
N-3	116	91	25	135	44	4.20	63.40
J-1	580	213	367	445	232	4.87	81.35
J-2	583	217	366	448	231	4.93	82.15
J-3	586	220	366	451	231	4.93	82.80

Note: N stand for non-waxy, and J stand waxy.

Table S4. Summary of transcriptome data during grain filling in foxtail millet.

Sample	Raw Data		Valid Data		Valid Ratio(reads)	Q20%	Q30%
	Read	Base	Read	Base			
J_3_1_15d	40877958	6.13G	39898540	5.98G	97.60	99.80	96.97
J_3_2_15d	41503894	6.23G	40297390	6.04G	97.09	99.80	96.82
J_3_3_15d	39686156	5.95G	39316050	5.90G	99.07	99.92	95.70
J_3_1_1d	42137904	6.32G	41045510	6.16G	97.41	99.19	96.00
J_3_2_1d	40207482	6.03G	39119660	5.87G	97.29	99.85	97.41
J_3_3_1d	39009590	5.85G	38631326	5.79G	99.03	99.92	95.56
J_3_1_35d	40701930	6.11G	37806210	5.67G	92.89	98.76	93.50
J_3_2_35d	41002102	6.15G	39699312	5.95G	96.82	99.85	96.89
J_3_3_35d	36085134	5.41G	35391144	5.31G	98.08	99.82	94.60
N_17_1_15d	40210574	6.03G	39159752	5.87G	97.39	99.21	96.06
N_17_2_15d	40483534	6.07G	39329392	5.90G	97.15	99.82	96.96
N_17_3_15d	41263752	6.19G	40003000	6.00G	96.94	98.99	95.73
N_17_1_1d	40878738	6.13G	39768992	5.97G	97.29	99.87	97.51
N_17_2_1d	40939326	6.14G	39164546	5.87G	95.66	99.84	96.08
N_17_3_1d	36741598	5.51G	35270014	5.29G	95.99	99.06	94.82
N_17_1_35d	40395280	6.06G	39191766	5.88G	97.02	99.09	95.92
N_17_2_35d	38452886	5.77G	37179826	5.58G	96.69	99.01	95.65
N_17_3_35d	38184788	5.73G	36646934	5.50G	95.97	98.87	95.52

Note: N stand for non-waxy, and J stand waxy.

Table S5. Summary of miRNA data during grain filling in foxtail millet.

Sample_ID	Total_Reads	Total_Bases	A%	T%	C%	G%	Q20%	Q30%	GC%
J_3_2_1d	11248588	573677988	25.27	23.37	24.01	27.34	98.94	96.45	51.36
J_3_2_35d	12033763	613721913	24.00	22.35	24.96	28.69	99.05	96.99	53.65
J_3_2_15d	12798645	652730895	24.18	21.59	25.78	28.44	99.02	96.93	54.23
J_3_3_35d	9331709	475917159	24.09	21.76	26.03	28.12	98.85	96.28	54.15
J_3_1_35d	11934957	608682807	24.43	22.92	24.62	28.03	99.01	96.75	52.65
J_3_3_1d	9522788	485662188	25.10	22.72	24.45	27.73	98.80	96.1	52.18
J_3_1_1d	10668386	544087686	24.67	21.95	25.11	28.27	98.94	96.6	53.38
J_3_3_15d	11064441	564286491	23.74	21.74	26.06	28.46	98.91	96.45	54.52
J_3_1_15d	11660229	594671679	24.06	21.88	25.47	28.59	99.05	96.88	54.06
N_17_3_35d	10487230	534848730	23.44	21.33	26.38	28.85	99.03	96.88	55.23
N_17_3_1d	12556561	640384611	25.02	22.67	24.81	27.51	98.94	96.6	52.31
N_17_3_15d	12021896	613116696	23.96	21.74	25.58	28.72	99.04	96.86	54.30
N_17_2_35d	11181845	570274095	23.17	21.61	26.65	28.57	99.07	97.03	55.22
N_17_2_1d	12303253	627465903	25.22	22.32	24.74	27.72	98.99	96.86	52.46
N_17_2_15d	9654033	492355683	24.39	21.98	25.19	28.44	98.74	95.95	53.63
N_17_1_35d	11002308	561117708	23.80	21.58	26.26	28.36	98.86	96.46	54.62
N_17_1_15d	12172866	620816166	24.43	21.93	24.97	28.67	98.90	96.54	53.64
N_17_1_1d	13259047	676211397	24.80	22.99	24.53	27.68	98.88	96.22	52.21

Note: N stand for non-waxy, and J stand waxy.

Table S6. Differentially expressed miRNAs were identified at 1DAA.

Index	miR_name	miR_seq	up/down	fold_change	log2fc	p-value	N_1d(mean)	J_1d(mean)
1	PC-3p-78123_153	AACAAGTGGACTAAAGTTTGGACT	up	inf	inf	3.56E-03	0	94
2	ssl-MIR166a-p5_1ss12TA	AATGGAGGCTGATCCAAGATC	up	3.34	1.74	1.40E-02	8	26
3	ptc-miR6478_R+2_1ss21GA	CCGACCTTAGCTCAGTTGGTAGA	down	0.62	-0.68	1.57E-02	1,021	636
4	PC-3p-210954_34	AGGGGAAAAAGA-GAAAAAAATGAAC	up	inf	inf	1.96E-02	0	17
5	PC-5p-403768_10	AGTAACACCAACCGGGACTAAAGG	down	-inf	-inf	1.97E-02	4	0
6	PC-3p-336052_14	AAAAGTAGGGTGTTAAAGTTTAAC	down	-inf	-inf	2.05E-02	3	0
7	PC-3p-266660_22	AACTATGAACCTAGAAATGCC	up	inf	inf	2.62E-02	0	4
8	PC-5p-320836_16	ACCCATTAGTACCGGATGGAGCCT	down	-inf	-inf	2.64E-02	4	0
9	PC-5p-152118_58	AGAAGATCGCTATTTGTTTGAGCT	up	3.49	1.80	2.86E-02	3	11
10	PC-5p-213878_33	ATACACCCTCATAAACTTTAGGAC	down	-inf	-inf	3.10E-02	3	0
11	mtr-MIR171b-p5_1ss21TC	AGGTATTGGCGCGCCTCAATC	up	1.98	0.98	3.20E-02	79	157
12	PC-3p-79731_148	AGACTTAGAAAAATCAAAACGACC	up	inf	inf	3.26E-02	0	43
13	PC-5p-304496_17	ATTTAGTACCGGGTGAAGTCTCCA	down	-inf	-inf	3.43E-02	4	0
14	mtr-miR171d_R+1	TGATTGAGCCGTGCCAATATCT	up	1.43	0.52	3.76E-02	9	13
15	PC-3p-181065_44	ATTTTCAACCGGGACTAAACACCT	down	-inf	-inf	3.93E-02	12	0
16	mtr-miR166e-5p_R+1_2ss10GT19AG	GGAATGTTGTCTGGCTCGGGGG	up	1.96	0.97	3.99E-02	9	17
17	PC-3p-183755_43	TGTTTCACTCCAATCCTCTTC	down	-inf	-inf	4.01E-02	6	0
18	ath-miR169a-3p_L+2	TCGGCAAGTTGTCCTTGGCTAC	up	1.29	0.37	4.04E-02	3	3
19	gma-MIR10195-p3_2ss1TA18CA	AAAAGATTGGAGAATGGA	down	-inf	-inf	4.11E-02	4	0
20	PC-5p-241954_27	ACCAACCGGGACAAAAGGCCAACT	down	-inf	-inf	4.14E-02	14	0
21	PC-5p-315771_16	AGTACCGTGTGGAACCTCCACCCA	down	-inf	-inf	4.41E-02	6	0
22	mtr-miR398b_1ss20TC	TGTGTTCTCAGGTCGCCCCCG	up	2.06	1.04	4.41E-02	32	65
23	mes-miR482b_2ss12GT21GT	TTCCCAATGTCTCCCATTCCTA	up	2.14	1.10	4.78E-02	10	20
24	PC-3p-216611_32	AAAATCTATGAATCTCAAAAAGCC	up	4.99	2.32	4.82E-02	2	9
25	PC-3p-203177_36	AAAAGTTATGAATCTAGAAAAGCC	up	3.66	1.87	4.90E-02	2	9
26	ath-MIR169a-p3_1ss1GC	CATCGGCAAGTTGTCCTTGGC	up	1.32	0.40	4.94E-02	103	137
27	PC-3p-226024_30	ATACAAGCTGTCTGTTTCACTGTC	up	inf	inf	5.21E-02	0	10
28	ppe-miR530_L-2R+2	TGCATTTGCACCTGCACCTCC	up	4.76	2.25	5.51E-02	72	345
29	PC-5p-158348_54	TTAGTACCGGTTGGAGCTCCCAAT	up	2.89	1.53	6.61E-02	2	7
30	PC-5p-415123_10	ATTTGTACTGGTGAGCTTCTTAAC	up	inf	inf	6.64E-02	0	8
31	PC-5p-92372_121	ATAGTACCGGTTTGGAGCCTCAGC	up	1.72	0.78	7.82E-02	12	20
32	mtr-miR167b-5p_R-1	TGAAGCTGCCAGCATGATCT	up	1.43	0.51	7.83E-02	167	238
33	PC-5p-300244_18	AACGCCAACCAGGACAAAAGCTCC	down	-inf	-inf	7.85E-02	4	0
34	PC-3p-300244_18	AACGCCAACCAGGACAAAAGCTCC	down	-inf	-inf	7.85E-02	4	0
35	PC-3p-87639_130	ATCATGCATGACAGCCTCATT	up	3.34	1.74	8.21E-02	8	27
36	mtr-miR164a_1ss21AT	TGGAGAAGCAGGGCACGTGCT	up	1.48	0.57	8.34E-02	139	206
37	ath-miR8175_L+1_1ss18GA	CGATCCCCGGCAACGGCACCA	down	-inf	-inf	8.43E-02	6	0
38	mtr-miR398a-3p	TGTGTTCTCAGGTCACCCCTT	up	1.69	0.75	8.46E-02	8	13
39	mtr-miR156i-3p_L-1_1ss9TA	GCTCACTACTCTTTCTGTCATC	up	1.52	0.60	8.54E-02	7	10
40	PC-3p-205674_35	AAAACTTATGAACCTAGAAATGCC	up	6.39	2.68	9.11E-02	1	9
41	PC-3p-142227_64	AGTTTAGGACCAGTTGGTGTTACC	up	2.59	1.37	9.19E-02	5	14
42	PC-5p-47413_301	AGTACCGGACCTAACGGCTAGTCC	up	2.05	1.03	9.34E-02	13	27
43	PC-3p-127455_75	ATATCTATGAATCTAAAAAGT	up	3.81	1.93	9.38E-02	2	7
44	PC-3p-225361_30	GACCTTTAGTACCGGTTGGAGCCT	down	0.17	-2.53	9.61E-02	8	1
45	aly-miR396b-5p_R+2	TTCCACAGCTTTCTTGAACTTTC	down	0.24	-2.08	9.70E-02	7	2
46	PC-5p-101603_105	GTTCTCCTCAAACTACTTCACT	up	2.50	1.32	9.79E-02	4	10

Table S7. Differentially expressed miRNAs were identified at 15DAA.

Index	miR_name	miR_seq	up/down	FC	log2FC	p-value	N_15d(mean)	J_15d(mean)
1	ath-miR8175_1ss2AT	GTTCCCCGGCAACGGCGCCA	down	0.71	-0.50	3.03E-03	70	49
2	PC-5p-57753_231	AAAGACACACTCGGCGAAATGGGC	down	0.39	-1.37	3.47E-03	83	32
3	PC-3p-140062_66	ACTTAATTTGGGACGGAGGGA	up	inf	inf	5.24E-03	0	7
4	mtr-miR156b-5p_L+1_1ss15TC	TTGACAGAAGAGAGCGAGCAC	down	0.50	-0.99	6.38E-03	153	77
5	PC-5p-4326_2827	AGGATTAGATGGAACTGAACC	up	inf	inf	6.59E-03	0	5
6	mtr-miR164a	TGGAGAAGCAGGGCACGTGCA	up	1.37	0.45	1.20E-02	10,357	14,197
7	sly-miR164b-3p_1ss20AT	CACGTGTTCTCCTTCTCCATC	down	-inf	-inf	1.21E-02	12	0
8	PC-5p-175590_46	ACTTTGCCGAGTGCACCCAGA	down	-inf	-inf	1.24E-02	6	0
9	mtr-miR167a	TGAAGCTGCCAGCATGATCTA	down	0.83	-0.26	1.84E-02	332	277
10	ptc-miR169i_R+2_1ss21GA	TAGCCAAGGATGACTTGCCTACA	down	0.16	-2.60	1.86E-02	10	2
11	aly-miR164a-3p_2ss6AG20AC	CACGTGCTCCCCTTCTCCACC	down	0.32	-1.65	1.89E-02	26	8
12	mtr-MIR167b-p3_2ss12GT21CT	AGATCATGTTGTAGCTTCACT	up	2.19	1.13	2.06E-02	55	120
13	mtr-miR166a_L+1R-1	TTCGGACCAGGCTTCATTCCC	up	1.13	0.17	2.34E-02	3,326	3,752
14	gma-miR482b-3p_1ss8TA	TCTTCCCAACACCTCCCATACC	down	-inf	-inf	2.40E-02	7	0
15	PC-5p-18269_929	ATTGAGCCGGGACTAAAGGGGACC	down	0.57	-0.80	2.55E-02	39	22
16	PC-3p-201769_37	ATCTCGGATAGGAGTAGAGCATGG	up	5.46	2.45	2.67E-02	1	5
17	PC-5p-70563_176	CTCTCGCCGGCGCGCGCGCTCG	down	0.23	-2.15	2.68E-02	7	2
18	mtr-miR172c-5p_2ss2TC7TC	GCAGCACCATCAAGATTCACA	up	1.76	0.82	2.74E-02	16	28
19	gma-miR172g_R+1	GCAGCACCATCAAGATTCACA	up	1.76	0.82	2.74E-02	16	28
20	ghr-miR482b_1ss11TC	TCTTGCCTACCCCACCCATGCC	down	-inf	-inf	3.08E-02	14	0
21	gma-MIR4357-p3_2ss5CT23AT	TGTATAAAACACATGACTCCTCT	down	-inf	-inf	3.10E-02	7	0
22	PC-5p-240402_27	CGCGAGACGAATATATTAAGCCTT	down	0.19	-2.40	3.11E-02	8	2
23	PC-5p-170382_48	CCTTTTGAAGTAACCCGTGGAGAA	down	-inf	-inf	3.25E-02	5	0
24	PC-5p-224869_30	TAGCTCCTAGTGATGTAGTCAGG	up	inf	inf	3.74E-02	0	4
25	PC-3p-61614_212	GTTCCCCTAAAAGCACTTCACG	up	3.03	1.60	3.76E-02	6	18

26	PC-3p-5632_2390	ATCTCGCCGAGGATGTTCTCC	up	1.32	0.40	4.16E-02	90	119
27	csi-miR166c-5p_L-1R+1_1ss19GA	GGAATGTTGTCTGGTTCAAGG	up	1.30	0.37	4.43E-02	180	233
28	mes-MIR171h-p5_1ss12TC	TGTTGGCTCGGCTCACTCAGA	up	1.49	0.58	4.51E-02	69	103
29	PC-3p-215312_33	TTAACACTCGGCAAAGGGGGCAGT	down	0.16	-2.68	4.53E-02	12	2
30	PC-3p-222_23321	TTTCCTATGCCATCTAATCCGA	down	0.68	-0.56	4.54E-02	443	301
31	ath-miR858b_1ss20TC	TTCGTTGTCTGTTCGACCTCG	down	0.81	-0.30	4.55E-02	21	17
32	PC-3p-54538_250	GAGAGAAGAATAAGCGGCTGCAGC	down	0.56	-0.85	4.89E-02	56	31
33	PC-3p-65317_195	ATCCCATGGCTGGGAACCAAGCAG	down	0.28	-1.86	4.90E-02	37	10
34	mtr-miR398a-3p	TGTGTTCTCAGGTCACCCCTT	down	0.62	-0.68	5.06E-02	12	8
35	ath-miR156b-3p_L-1_2ss8CT14TC	GCTCACTTCTCTCTCTGTCAGT	down	0.23	-2.15	5.14E-02	29	7
36	ppe-MIR156c-p3_1ss22CT	GCTCACTTCTCTCTCTGTCAGT	down	0.23	-2.15	5.14E-02	29	7
37	PC-5p-200162_37	AAGTGTCTGGACGGGGCACTCGGC	up	inf	inf	5.18E-02	0	4
38	lja-MIR167a-p3_1ss13AG	GGTCATGCTGCGGCAGCCTCACT	down	-inf	-inf	5.32E-02	11	0
39	PC-5p-177089_45	CGTGAGACAACTCTATTAAGGCTA	up	4.65	2.22	5.37E-02	1	7
40	PC-5p-18284_928	ACGGTAGGCACTCGGCAAACACCA	down	0.54	-0.90	5.41E-02	155	83
41	PC-5p-221_23413	CGATATAGCTCGGTGGCGTGTCTG	down	0.61	-0.71	5.48E-02	1,706	1,042
42	PC-5p-111983_91	AGATCGGCACCGGAGGGCTGA	up	4.42	2.15	5.53E-02	1	6
43	gma-miR169j-5p_R-1_1ss20GT	TAGCCAAGAATGACTTGCCT	down	0.36	-1.46	5.61E-02	7	3
44	cas-miR5139_L-1	AACCTGGCTCTGATACCA	up	1.49	0.58	5.62E-02	61	91
45	csi-MIR156f-p5_2ss18AG21CT	AGAAGAGAGAGAGTACAGCCT	up	1.37	0.45	5.78E-02	1,712	2,344
46	gma-MIR319a-p5_1ss18TC	AGCTGCCGACTCATTCACCCA	up	1.52	0.61	5.86E-02	297	452
47	PC-3p-126535_76	AAGTTTAAGGACCCAGATGCC	up	3.02	1.60	6.05E-02	5	15
48	PC-3p-131819_72	CAACTCAGACATGTCACCGATCTA	up	6.62	2.73	6.09E-02	2	11
49	fve-miR396e_L-1R+1	TCCACAGGCTTTCTTGAACTG	up	1.23	0.30	6.14E-02	3,281	4,035
50	PC-3p-112503_91	TTTAACACTCGGCAAAGGGGGCAC	down	0.33	-1.61	6.15E-02	18	6
51	csi-miR160c-3p_2ss7GA16TA	GCGTGCAAGGAGCCAAGCATG	up	inf	inf	6.43E-02	0	4
52	mdm-MIR166c-p5_2ss16TC17CG	GGAATGTTGTCTGGTCGGAGA	up	1.35	0.43	6.62E-02	18	25
53	mtr-miR160a	TGCCTGGCTCCCTGTATGCCA	down	0.80	-0.33	6.64E-02	727	580
54	mtr-miR408-3p_1ss1AC	CTGCACTGCCTCTTCCCTGGC	down	0.37	-1.42	6.67E-02	60	22
55	mtr-miR166e-5p_R+1_2ss10GT19AG	GGAATGTTGTCTGGCTCGGGGG	down	0.71	-0.49	6.78E-02	30	21
56	PC-5p-175621_46	GATGGAGGCTAAATGACGAGACAA	down	0.24	-2.05	6.82E-02	8	2
57	PC-3p-96070_114	AATAGATTCGTCTCGATTTAGCCT	up	inf	inf	6.85E-02	0	10
58	mdm-miR171i	TGAGCCGAACCAATATCACTC	up	1.26	0.34	7.40E-02	357	451
59	rgl-miR5139_L+2	CGAAACCTGGCTCTGATACCA	up	1.34	0.42	7.53E-02	16	22
60	cas-miR5139_L+2	CGAAACCTGGCTCTGATACCA	up	1.34	0.42	7.53E-02	16	22
61	ath-miR8175_L-2_1ss17GT	TCCCCGGCAACGGCTCCA	down	0.24	-2.04	8.02E-02	7	2
62	mtr-miR171d	TGATTGAGCCGTGCCAATATC	down	0.63	-0.66	8.03E-02	579	367
63	PC-3p-76633_157	ATTTTCAACCGGAACAAAACGCCC	down	-inf	-inf	8.07E-02	11	0
64	PC-3p-90439_124	TTAACACTCGGCAAAGGGGGCACT	down	0.42	-1.26	8.13E-02	23	10
65	mtr-miR398b_1ss20TC	TGTGTTCTCAGGTCGCCCCCG	down	0.42	-1.24	8.14E-02	28	12
66	PC-5p-101603_105	GTTCTCCTCAAACTACTTCACT	up	2.22	1.15	8.37E-02	11	24
67	mtr-miR166g-5p	GGAATGTTGTCTGGCTCGAGG	up	1.25	0.32	8.54E-02	596	745
68	mtr-miR166a_1ss10GA	TCGGACCAGACTTCATTCCCC	down	0.85	-0.23	8.61E-02	282	241
69	PC-5p-32876_479	TGTGTTCCTCTGTTTGGCGCTAAAG	down	0.48	-1.07	8.73E-02	79	38
70	mtr-miR160c	TGCCTGGCTCCCTGAATGCCA	down	0.77	-0.37	9.09E-02	57	44
71	gma-MIR4391-p5_2ss16GA17AT	AAGAACTAAGAAGAAATACAATT	down	0.55	-0.85	9.33E-02	8	4
72	gma-MIR4391-p3_2ss16GA17AT	AAGAACTAAGAAGAAATACAATT	down	0.55	-0.85	9.33E-02	8	4
73	mtr-miR167a_R+1_1ss21AG_2	TGAAGCTGCCAGCATGATCTGG	up	1.35	0.43	9.46E-02	53	72
74	mtr-miR319a-5p_1ss6TG	AGAGCGTCCTTCAGTCCACTC	down	0.74	-0.44	9.79E-02	69	51

Table S8. Differentially expressed miRNAs were identified at 35DAA.

Index	miR_name	miR_seq	up/down	FC	log2FC	p-value	N_35d(mean)	J_35d(mean)
1	PC-3p-176398_46	TCTAAACATAGGGCTAAAGTT	up	inf	inf	2.95E-04	0	3
2	mtr-MIR167b-p3_2ss12GT21CT	AGATCATGTTGTAGCTTCACT	up	7.22	2.85	6.22E-04	9	62
3	mtr-miR166c_1ss20TC	TCGGACCAGGCTTCATTCCCC	down	0.64	-0.64	7.88E-04	310,269	198,976
4	mtr-miR166a	TCGGACCAGGCTTCATTCCCC	down	0.64	-0.64	7.88E-04	310,269	198,976
5	mtr-miR166a_2ss16TA21CT	TCGGACCAGGCTTCAATCCCT	down	0.70	-0.52	1.30E-03	9,132	6,367
6	PC-3p-131819_72	CAACTCAGACATGTCACCGATCTA	up	15.65	3.97	1.42E-03	2	31
7	ath-miR8175_L+4_1ss6AT	GTTCGTTCCCCGGCAACGGCGCCA	down	0.50	-0.99	2.08E-03	111	56
8	PC-5p-1076_6697	TCAGGAGAGATGACACCGACG	up	2.18	1.12	4.00E-03	221	482
9	PC-3p-54538_250	GAGAGAAGAATAAGCGGCTGCAGC	up	inf	inf	5.55E-03	0	5
10	vvi-MIR169g-p5	ATGGTGCAGCCAAGGATGACT	up	2.60	1.38	5.86E-03	44	113
11	mtr-miR166c	TCGGACCAGGCTTCATTCCTC	down	0.76	-0.39	6.26E-03	3,836	2,934
12	mtr-miR166a_1ss10GA	TCGGACCAGACTTCATTCCCC	down	0.66	-0.61	6.59E-03	1,086	713
13	gma-miR399i	TGCCAAAGGAGAATTGCCCTG	up	4.18	2.06	6.59E-03	336	1,404
14	ptc-miR6478_1ss21GA	CCGACCTTAGCTCAGTTGGTA	up	1.45	0.54	7.10E-03	351	511
15	PC-5p-13606_1222	AATGATGTCCGGTCCGAAGGC	up	2.79	1.48	7.17E-03	43	119
16	PC-3p-7990_1876	TTCAGTTTCCTCTAATATCTCA	up	inf	inf	8.43E-03	0	4
17	mtr-miR167b-5p_R+1_2	TGAAGCTGCCAGCATGATCTGT	up	1.86	0.90	8.61E-03	63	117
18	mtr-miR167a_R+1_1ss21AG_1	TGAAGCTGCCAGCATGATCTGA	up	1.86	0.90	8.61E-03	63	117
19	mtr-miR167b-5p_R+1_1	TGAAGCTGCCAGCATGATCTGA	up	1.86	0.90	8.61E-03	63	117
20	ppe-miR156c_R+1	TGACAGAAGAGAGTGAGCACG	down	0.69	-0.55	9.84E-03	29	20
21	ptc-miR169i_R+1_1ss14CT	TAGCCAAGGATGATTTGCCTGT	up	8.38	3.07	1.03E-02	1	8
22	PC-5p-5976_2302	ATTTCGTGGAATAACCCTTCAAGA	up	3.52	1.82	1.06E-02	117	414
23	ath-miR8175_L+3_1ss5AT	TTCGTTCCCCGGCAACGGCGCCA	down	0.35	-1.51	1.08E-02	77	27
24	mtr-miR167a_L+1R+1_1ss22AG	CTGAAGCTGCCAGCATGATCTGA	up	1.98	0.99	1.15E-02	7	14
25	PC-3p-83583_139	ACACAAACCGAGACTAAAGGGTTC	up	inf	inf	1.16E-02	0	7
26	PC-5p-124079_79	CTTAGTATTGGGTAGAGCCTC	down	-inf	-inf	1.20E-02	13	0
27	ath-miR8175_L+1_1ss3AT	CGTTCCCCGGCAACGGCGCCA	down	0.35	-1.52	1.40E-02	283	99
28	csi-MIR156f-p5_2ss18AG21CT	AGAAGAGAGAGAGTACAGCCT	up	1.93	0.95	1.42E-02	173	335
29	mtr-MIR164c-p5_1ss1TN	NGGAGAAGCAGGGCACGTGCA	up	6.59	2.72	1.48E-02	2	12
30	PC-3p-222_23321	TTTCCTATGCCATCTAATCCGA	up	1.55	0.64	1.53E-02	384	596
31	mtr-miR156a_R-1	TGACAGAAGAGAGAGAGCAC	down	0.41	-1.29	1.57E-02	29	12
32	ptc-miR399b	TGCCAAAGGAGATTTGCCCGG	up	inf	inf	1.79E-02	0	4
33	ath-miR8175_L-1_1ss2AC	CTCCCCGGCAACGGCGCCA	down	0.26	-1.93	1.84E-02	10	3
34	mdm-miR171i	TGAGCCGAACCAATATCACTC	up	1.66	0.73	2.10E-02	235	389
35	gma-miR6300_R+6	GTCGTTGTAGTATAGTGGTGAGTA	up	3.20	1.68	2.26E-02	10	32

36	PC-3p-141686_64	AGTGCGCGACAGAAAACACTCGGC	up	inf	inf	2.27E-02	0	4
37	PC-5p-236305_28	TTTTGGCTTTTTTAGATACATATG	up	inf	inf	2.33E-02	0	4
38	ath-miR8175_L-1_1ss2AG	GTCCCCGGCAACGGCGCCA	down	0.27	-1.91	2.45E-02	9	2
39	ptc-miR6478_R+2_2ss10GA21GA	CCGACCTTAACTCAGTTGGTAGA	up	1.65	0.72	2.45E-02	20	34
40	mtr-miR166a_1ss14CA	TCGGACCAGGCTTAATTCCCC	down	0.54	-0.89	2.65E-02	29	16
41	PC-3p-54_92727	TTAGCGTCAAGAGACGAACACACT	up	2.72	1.44	2.75E-02	1,452	3,948
42	mtr-miR164a	TGGAGAAGCAGGGCACGTGCA	up	1.40	0.49	2.81E-02	5,359	7,509
43	mtr-MIR171b-p5_1ss21TC	AGGTATTGGCGCGCCTCAATC	up	3.86	1.95	3.02E-02	5	19
44	gma-miR6300_R+7	GTCGTTGTAGTATAGTGGTGAGTAT	up	3.00	1.58	3.31E-02	5	14
45	ptc-miR6478_2ss10GA21GA	CCGACCTTAACTCAGTTGGTA	up	2.42	1.28	3.37E-02	7	16
46	PC-5p-183416_43	AAAATGTAGGTCGTTTTCGCA	up	5.88	2.56	3.41E-02	1	5
47	PC-3p-100632_107	ATCTCGCCGAGGATGTTCTCCG	up	3.25	1.70	3.43E-02	3	11
48	mtr-miR166a_1ss21CA	TCGGACCAGGCTTCATTCCCA	down	0.88	-0.18	3.72E-02	7,223	6,383
49	ath-miR8175_L-2	TCCCCGGCAACGGCGCCA	down	0.51	-0.97	3.80E-02	1,508	770
50	ath-miR827_2ss15AG21TA	TTAGATGACCATCAGCAAACA	up	1.82	0.87	3.94E-02	5,797	10,564
51	ath-miR8175_1ss2AT	GTTCCCCGGCAACGGCGCCA	down	0.36	-1.49	3.98E-02	74	26
52	ath-miR8175_L+1_1ss3AG	CGGTCCCCGGCAACGGCGCCA	down	0.32	-1.64	4.04E-02	32	10
53	vvi-miR399b_L+2R-2	TCTGCCAAAGGAGAGTTGCCC	up	2.33	1.22	4.11E-02	8	18
54	gra-MIR166d-p5_1ss12TC	TCGGACCAGGCCTCATTCCCA	up	inf	inf	4.49E-02	0	7
55	ssl-MIR166a-p5_1ss12TA	AATGGAGGCTGATCCAAGATC	up	3.91	1.97	4.61E-02	10	40
56	ppe-miR530_L-2R+2	TGCATTTGCACCTGCACCTCC	up	3.68	1.88	4.65E-02	54	198
57	mtr-miR319a-5p_1ss6TG	AGAGCGTCCTTCAGTCCACTC	up	2.74	1.45	4.87E-02	59	161
58	PC-3p-142121_64	TTATAACCCGGTACTAAAGGT	up	inf	inf	5.10E-02	0	5
59	mtr-miR393a_L+1R-1	CTCCAAAGGGATCGCATTGAT	up	1.75	0.81	5.23E-02	541	948
60	nta-MIR6156-p3_2ss10AC19TC	TGAATAACACCAGATGCCCTT	down	0.31	-1.68	5.26E-02	6	2
61	PC-3p-104142_102	AATTTTGGGACAGAGGTAGTA	up	2.94	1.56	5.36E-02	4	12
62	PC-3p-104350_101	TTTAGTACCGGTTGGAGCTCCCAA	down	0.58	-0.78	5.49E-02	13	8
63	PC-3p-224043_31	TTAATAGATTCGTCTCGTTTT	up	inf	inf	5.66E-02	0	10
64	PC-3p-132381_71	TAAAATCGAACCTCTTTCATG	up	4.39	2.14	5.69E-02	1	4
65	ath-MIR169a-p3_1ss1GC	CATCGGCAAGTTGTCCTTGGC	up	1.60	0.68	6.10E-02	130	208
66	mtr-miR172c-5p_2ss2TC7TC	GCAGCACCATCAAGATTCACA	up	4.74	2.24	6.32E-02	5	24
67	gma-miR172g_R+1	GCAGCACCATCAAGATTCACA	up	4.74	2.24	6.32E-02	5	24
68	PC-5p-39346_383	TTTTACTACCCGGAACAATCC	down	0.74	-0.44	6.33E-02	24	18
69	mdm-MIR11004-p5_2ss5GC17TC_1	GGGACGTGGTGGTTGACG	down	0.66	-0.61	6.33E-02	28	18
70	mdm-MIR11004-p5_2ss5GC17TC_2	GGGACGTGGTGGTTGACGG	down	0.66	-0.61	6.33E-02	28	18
71	ath-miR8175_L-1_1ss2AT	TTCCCCGGCAACGGCGCCA	down	0.39	-1.36	6.68E-02	173	67
72	mtr-MIR2592bi-p3_2ss8TC19AT	ATTCCCACTGTCCCTGTCT	down	0.50	-1.00	6.71E-02	48	24
73	PC-3p-227498_30	TTCATCTCGCCGAGGATGTTCTCC	up	4.17	2.06	6.86E-02	1	4
74	mtr-MIR2592bj-p3_2ss10TC17AT	TCCCACTGTCCCTGTCTA	down	-inf	-inf	7.43E-02	8	0
75	mtr-MIR2592bi-p3_2ss6TC17AT	TCCCACTGTCCCTGTCTA	down	-inf	-inf	7.43E-02	8	0
76	fve-miR396e_L-1R+1	TCCACAGGCTTTCTTGAACTG	up	3.26	1.70	7.49E-02	723	2,352
77	PC-3p-36270_425	TTCGCCTCGGCGAGGATGTTCTC	up	2.79	1.48	7.59E-02	5	14
78	PC-5p-40695_367	GGATTAGGTGCTTGGGAAAGT	up	2.36	1.24	7.85E-02	7	17
79	ptc-miR399a_1ss11GA	TGCCAAAGGAAATTTGCCCCG	up	inf	inf	7.92E-02	0	12
80	mtr-miR396b-5p_1ss21GT	TTCCACAGCTTTCTTGAACTT	up	2.97	1.57	8.00E-02	109	325
81	mtr-miR156b-5p_1ss8AC	TGACAGACGAGAGTGAGCAC	down	0.66	-0.59	8.07E-02	114	76
82	PC-3p-5632_2390	ATCTCGCCGAGGATGTTCTCC	up	2.07	1.05	8.46E-02	189	392
83	PC-3p-118128_85	TAAAATCGAACCTCTTTCATGA	up	2.56	1.36	8.79E-02	3	8
84	mtr-miR166a_1ss4GA	TCGAACCAGGCTTCATTCCCC	down	0.58	-0.79	8.85E-02	18	11
85	mtr-miR156b-5p_L+1_1ss15TC	TTGACAGAAGAGAGCGAGCAC	down	0.74	-0.44	8.93E-02	1,136	839
86	ath-miR164c-5p_2ss13GA20CA	TGGAGAAGCAGGACACGTGAG	down	0.41	-1.28	9.34E-02	48	20
87	lus-miR159b_R+1_1ss21CT	TTTGGATTGAAGGGAGCTCTTT	down	0.73	-0.45	9.39E-02	508	373
88	ath-miR8175_L+1	CGATCCCCGGCAACGGCGCCA	down	0.53	-0.92	9.41E-02	1,056	559
89	mtr-MIR2592bi-p3_1ss8TC	ATTCCCACTGTCCCTGTC	down	0.35	-1.50	9.79E-02	43	15
90	mtr-miR172b	AGAATCTTGATGATGCTGCAT	up	28.98	4.86	9.86E-02	2	54

Table S9. MiRNAs regulating genes related to starch synthesis.

Query	Transcript	GnenID	TStart	TStop
ctr-MIR166-p5_2ss11CG18CT	Seita.4G022400.1.v2.2	Seita.4G022400.v2.2	1129	1147
PC-5p-221_23413	Seita.4G099700.1.v2.2	Seita.4G099700.v2.2	2264	2287
ptc-miR169i_R+2_1ss21GA	Seita.9G129400.1.v2.2	Seita.9G129400.v2.2	2087	2109
mtr-miR162	Seita.1G179000.1.v2.2	Seita.1G179000.v2.2	1778	1799
